# Winter Bird Assemblages in Rural and Urban Environments: A National Survey

**DOI:** 10.1371/journal.pone.0130299

**Published:** 2015-06-18

**Authors:** Piotr Tryjanowski, Tim H. Sparks, Waldemar Biaduń, Tomasz Brauze, Tomasz Hetmański, Rafał Martyka, Piotr Skórka, Piotr Indykiewicz, Łukasz Myczko, Przemysław Kunysz, Piotr Kawa, Stanisław Czyż, Paweł Czechowski, Michał Polakowski, Piotr Zduniak, Leszek Jerzak, Tomasz Janiszewski, Artur Goławski, Leszek Duduś, Jacek J. Nowakowski, Andrzej Wuczyński, Dariusz Wysocki

**Affiliations:** 1 Institute of Zoology, Poznań University of Life Sciences, Wojska Polskiego 71C, 60–625, Poznań, Poland; 2 Department of Zoology and Invertebrate Ecology, John Paul II Catholic University of Lublin, Al. Kraśnicka 102, 20–718, Lublin, Poland; 3 Department of Vertebrate Zoology, Faculty of Biology and Environment Protection, Nicolaus Copernicus University, Lwowska 1, 87–100, Toruń, Poland; 4 Department of Zoology, Pomeranian University, Arciszewskiego 22b, 76–200, Słupsk, Poland; 5 Institute of Nature Conservation, Polish Academy of Sciences, Mickiewicza 33, 31–120, Kraków, Poland; 6 Department of Zoology and Landscaping, University of Technology and Life Sciences, Ks. A. Kordeckiego 20, 85–225, Bydgoszcz, Poland; 7 Przemysl Ornithological Society, Węgierska 6, 37–700, Przemyśl, Poland; 8 Upper Silesian Ornithological Society, pl. Jana III Sobieskiego 2, 41–902, Bytom, Poland; 9 Institute for Tourism and Recreation, State Higher Vocational School in Sulechów, Armii Krajowej Str. 51, 66–100, Sulechów, Poland; 10 Department of Environmental Protection and Management, Bialystok University of Technology, Wiejska 45a, 15–351, Białystok, Poland; 11 Department of Avian Biology & Ecology, Faculty of Biology, Adam Mickiewicz University, Umultowska 89, 61–614, Poznań, Poland; 12 Faculty of Biological Sciences, University of Zielona Góra, Prof. Z. Szafrana St. 1, 65–516, Zielona Góra, Poland; 13 Department of Teacher Training and Biodiversity Studies, University of Łódz, Banacha 1/3, 90–237, Łódź, Poland; 14 Department of Zoology, University of Natural Sciences and Humanities in Siedlce, Prusa 12, 08–110, Siedlce, Poland; 15 Institute of Nature Conservation, Polish Academy of Sciences, Lower-Silesian Field Station, Podwale 75, 50–449, Wrocław, Poland; 16 Department of Ecology & Environmental Protection, University of Warmia and Mazury in Olsztyn, Plac Łódzki 3, 10–727, Olsztyn, Poland; 17 Department of Vertebrate Anatomy and Zoology, University of Szczecin, Wąska 13, 71–412 Szczecin, Poland; Liverpool John Moores University, UNITED KINGDOM

## Abstract

Urban development has a marked effect on the ecological and behavioural traits of many living organisms, including birds. In this paper, we analysed differences in the numbers of wintering birds between rural and urban areas in Poland. We also analysed species richness and abundance in relation to longitude, latitude, human population size, and landscape structure. All these parameters were analysed using modern statistical techniques incorporating species detectability. We counted birds in 156 squares (0.25 km2 each) in December 2012 and again in January 2013 in locations in and around 26 urban areas across Poland (in each urban area we surveyed 3 squares and 3 squares in nearby rural areas). The influence of twelve potential environmental variables on species abundance and richness was assessed with Generalized Linear Mixed Models, Principal Components and Detrended Correspondence Analyses. Totals of 72 bird species and 89,710 individual birds were recorded in this study. On average (±SE) 13.3 ± 0.3 species and 288 ± 14 individuals were recorded in each square in each survey. A formal comparison of rural and urban areas revealed that 27 species had a significant preference; 17 to rural areas and 10 to urban areas. Moreover, overall abundance in urban areas was more than double that of rural areas. There was almost a complete separation of rural and urban bird communities. Significantly more birds and more bird species were recorded in January compared to December. We conclude that differences between rural and urban areas in terms of winter conditions and the availability of resources are reflected in different bird communities in the two environments.

## Introduction

Urban development is increasing across the globe, with major impacts on animal life-histories [1,2,3]. Ecological effects of urbanization have long been recognized, e.g. disturbance regimes, changes in light conditions, habitat distribution, predation pressure, and species composition [4,5,6,7]. In addition, urban environments support more anthropogenic food resources, and the climate of urban areas differs from that of nearby rural environments [6,8,9] due to the so-called urban heat island phenomenon [9].

Urban environments provide more stable and predictable food supplies, higher temperatures and reduced temperature variability [2,10,11]. Food may be more readily available in the proximity of humans during winter, thereby facilitating urbanization of wildlife, at least in sedentary and partially migratory species [12]. In consequence, survival in cities may be easier than in other habitats [13,14,15,16]. On the other hand, non-natural habitats, non-natural food resources, traffic related mortality and disease risk, may negatively impact birds living in urban environments [9,11]. The structure of habitats may be complex in some urban areas, which can be especially important in winter when birds may need to forage in different locations to meet energetic demands and find roosting sites [4,6,13]. However, to date, the majority of studies on the effect of urbanization, and comparisons of rural and urban avifauna, have only been carried out in the breeding season, and have focussed on local scales [3,4,17,18]. Therefore, knowledge about the large scale distribution and diversity of birds in winter appears crucial for understanding the effects of faster urbanization rates in recent decades [3,4].

The objectives of this study were to identify differences in bird communities between rural and urban areas in winter. Differences in species richness and population density between urban and nearby rural environments provide an estimate of the extent to which different species have adapted to the urban environment [17]. Obviously, some factors other than urbanization level (e.g. human disturbance, microclimate, difference in dispersal, predation pressure) may influence bird density, and may affect different ecological groups and particular species in different ways [19]. To reduce potential local effects we decided to carry out our study at a national scale, with study sites located throughout Poland. We paid special attention to the location and characteristics of study squares (e.g. the cover of different microhabitats) which might influence bird species richness and density during winter. Our focus on a large geographical area covers a wide range of winter environments, because in Poland there is a marked increase in winter severity from the north to the south (from the Baltic Sea to the Carpathian Mountains), and even more so from the west to the east (from Atlantic influence to a more continental climate; [20]). The severity of winter has been suggested as the main factor affecting winter bird communities in temperate zones [13,16,21].

Therefore, mainly due to the availability of additional food and thus improved survival, we predict higher species richness and population densities of birds in urban areas during winter than in the surrounding rural areas. Although this idea is simple it is surprising that, to the best of our knowledge, this has not been investigated in winter at so large a geographical scale.

## Material and Methods

### Ethics Statement

Since this was a purely observational study, no permission was required for fieldwork. We confirm that for all locations and activities no specific permission was necessary. We confirm that the field studies did not involve endangered or protected species, or the collection of, or sampling from, animals. The coordinates of the study locations are provided in S1 Table. Our study was carried out by direct observation of birds and the methods are described below. For this kind of study, i.e. observations in non-protected areas, it is not necessary in Poland to have approval from an Institutional Animal Care and Use Committee (IACUC) or equivalent animal ethics committee.

### Study areas

**Fig 1:**
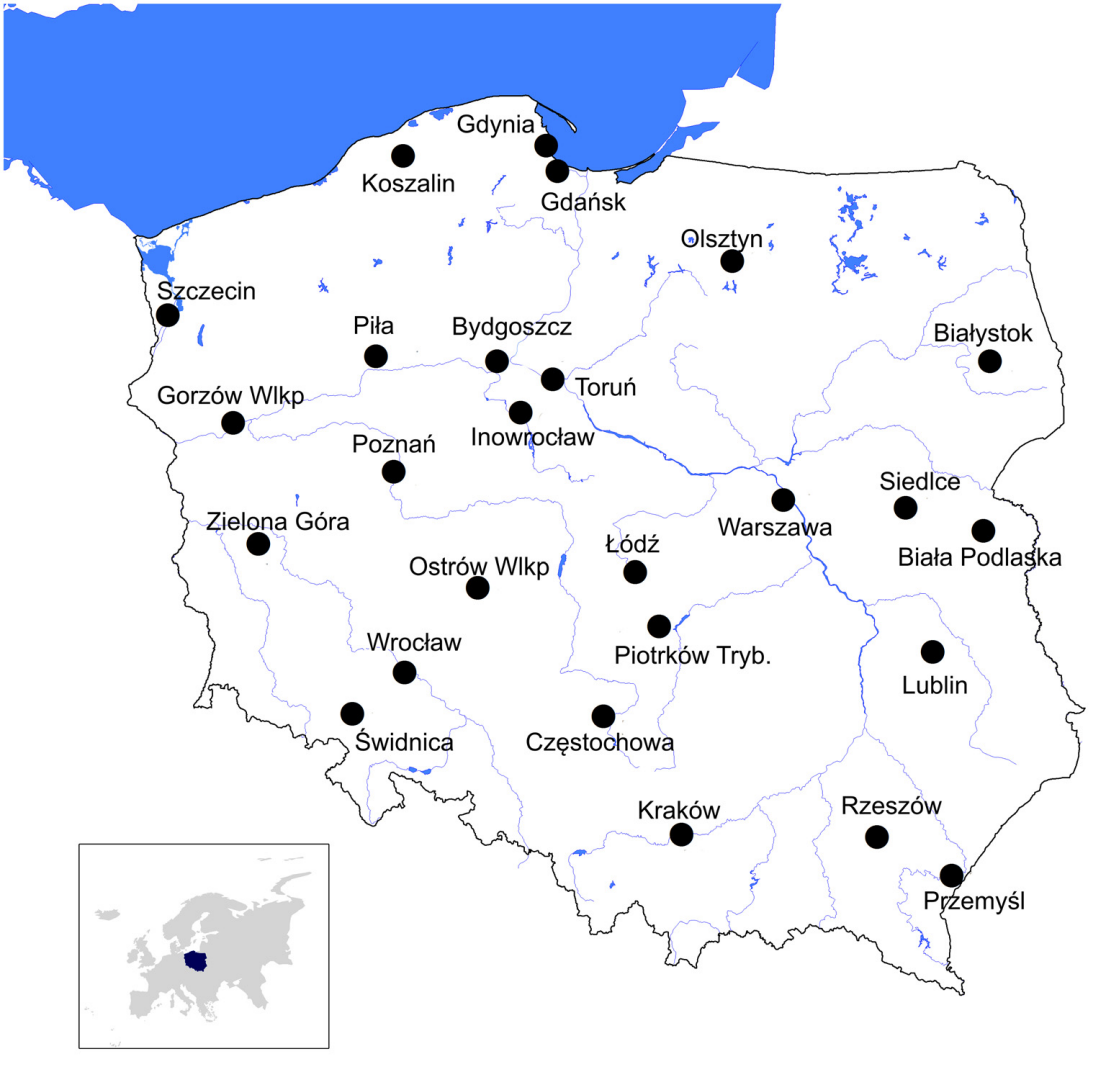
Location of the study areas. Location of the 26 paired areas used to study winter differences in birds between rural and urban environments in Poland.

Using the same methods, we recorded wintering birds in 26 towns and cities (hereafter called urban areas), each paired with a nearby rural area, across Poland (Fig 1; for more details see S1 Table). The study areas were chosen to cover all of Poland and span the entire Polish winter climate. Within each urban and rural area there were three square plots (25 ha) where birds were surveyed. Thus, the total number of squares was 156. The distance between paired rural and urban squares was 1–12 km. The benefit of this approach is that paired rural and urban study squares were characterised, as far as is practical, by similar climatic conditions. Squares were classified as urban or rural based on two criteria which both had to be met: (1) local authority designated as urban or rural (land management and policy in cities differs from that in rural districts); (2) squares in both environments had to include built up areas. For example, squares consisting only of arable land in urban local authorities were not considered. On average, each observer surveyed 1.81 ± 0.17 SE paired areas (range: 1–3), and all paired squares (urban-rural) were always visited by the same observer.

#### Field methods

Birds were counted twice in the winter of 2012/2013: firstly in December 2012 and repeated in January 2013. At this time of year, only truly wintering birds occur in Poland. As stated above, counts were carried out within three 0.25 km2 squares (500 × 500 m) in each of the urban areas, and in three 0.25 km2 squares within the neighbouring rural area, with surveys paired in time as closely as possible. The order of recording rural and urban squares was chosen randomly by observers. Birds were surveyed during favourable weather conditions (no snowfall or rain, wind below 4 m s-1) between 8:00 and 13:00. Single observers, with at least 10 years’ experience in counting wintering birds, walked in a *zig-zag* pattern in order to cover the entire square visually and to note bird vocalizations [13]. The duration of the survey was *ca* 2 hours for each square. Only birds exhibiting resting or foraging behaviour were included in the analysis. Hence, for example, high flying gulls, geese and corvids were ignored. The survey time ensured that birds were mainly foraging, and flights were rare. In some species, flights occurred between foraging and roosting places, however these occur mainly in the early morning and late afternoon and are thus outside the period devoted to fieldwork.

We measured the following environmental variables potentially affecting bird species richness (number of species) and abundance (number of individuals recorded in study squares):

1. type of environment: urban or rural;
2. cover (%) within the square of: trees, amenity grass, arable, fallow, meadows, buildings and roads, water;
3. number of bird feeders;
4. human population size in the urban area;
5. geographical latitude and longitude.

### Data handling and analysis

Cover variables were calculated with ImageJ software from detailed maps and aerial photos of the studied squares or directly in the field using a GPS. We used high resolution images, freely available from the National Data Base Geoportal (http://maps.geoportal.gov.pl/webclient/). Basic characteristics of the investigated urban areas are summarised in S1 Table, and their location within Poland is presented in Fig 1. For each square the total number (richness) of species, total number of birds (abundance) and Gini-Simpson Index of Diversity [22]) were calculated. The Gini-Simpson index gives the probability that two randomly chosen birds (individuals) from a community are not the same species. Thus, the higher values of this index the more diverse the community is. The index was moderately correlated with species richness (Spearman rs = 0.455, P<0.001, n = 312) but not with number of birds (Spearman rs = -0.072, P = 0.206, n = 312) which justified its use in the analyses.

Although detectability of species and of individuals in winter is relatively high [23,24,25], we also calculated the number of species and number of birds corrected for imperfect detection. The problem of detectability of species in our analyses was solved by using probabilistic methods for correcting species richness known as the Chao1 bias-corrected estimator of species richness [26,27]. Chao’s estimator is commonly used in ecological science and it was derived from the observation that rare species are undetectable because they are represented mostly by single individuals (singletons) or two individuals (doubletons). The formal equation for this index is: E(*S*) = *S*obs+ *n*1(*n*1-1)/ 2(*n*2+1), where: E(*S*) is the estimated number of species, *S*obs is the observed number of species, *n*1 is the number of singleton species, and *n*2 is the number of doubleton species [26]. A simulation study showed the superior performance (lower bias, higher precision and accuracy) of this index over many parametric methods (e.g. rarefaction curves) [28].

The corrected number of species in a square was calculated for each survey month separately in Spade software [29]. Then, to estimate the detectability of species we subtracted the recorded number of species from the estimated number of species for each square. This estimate indicates how many species remained undetected in a square and thus may be used to compare differences in species detectability between environments, months, and observers. The difference between observed and estimated numbers of species was analysed with generalized linear mixed models (see below).

We used two methods, proposed by Royle [30] and by Kendall et al. [31] and implemented in the Presence 6.1. software [32], to correct for imperfect detection of individuals. The Royle estimator [30] (contrary to many other methods based on presence-absence data and devoted to calculation of detectability) directly takes the number of individuals into account. However, the method assumes the population is closed, which might not be entirely true in winter. Violation of this assumption causes the estimated detection probability to be always lower than in reality and leads to excessive estimation of abundance, although this should still correctly represent differences between environments. In order to validate the Royle estimator, the unbiased estimator of detectability proposed by Kendall [31] was used. This method relaxes the closure assumption within a season by permitting staggered entry and exit times for the species of interest at each site (square). However, this method requires at least three surveys to estimate confidence intervals but we were only able to perform two surveys (December and January). Thus, we calculated the correlation coefficient between Royle and Kendall estimators which was statistically significant (S1 Fig). Moreover, we validated the Royle estimator by plotting species-estimates of detectability against their body sizes (= body length). Detectability is usually positively related to body size [33] and we found that this was also the case in our study (S2 Fig). Thus, we used the Royle estimator to correct abundances for imperfect detection in our data.

The detection probabilities of species and individuals were calculated for each environment and survey (December and January) separately. We conducted and present two sets of analyses—with and without corrections for detectability. Although corrected and uncorrected data were significantly correlated (S3 and S4 Figs) the results of statistical analyses were different. Therefore, we present only results based on corrected data.

Before formal testing of the effects of environmental variables on species richness, abundance and diversity index we had to perform data reduction. Since there were seven habitat composition variables which were correlated with each other (S2 Table), we used Principal Components Analysis (PCA) to calculate a reduced number of independent variables. We included longitude and latitude as supplementary variables in PCA to obtain ordination scores of environmental variables which were not correlated with geographical coordinates. The first two principal components explained 54% of the variability in habitat cover variables (Table 1, S5 Fig). The first principal component (PCA1, eigenvalue = 1.952) was associated with a gradient from built up areas to open agricultural habitats. The second principal component (PCA2, eigenvalue = 1.190) described a gradient from semi-natural grasslands to intensively managed amenity grasses (Table 1, S5 Fig).

**Table 1:** Results of the Principal Components Analysis (PCA) performed on the correlation matrix of the environmental variables describing cover of different habitat types.

Variable	Comm. (%)	PCA1	PCA2
Buildings and roads	70.3	**-0.831**	0.113
Fallow	49.8	**0.704**	0.046
Arable	63.5	**0.615**	**0.507**
Water	20.5	0.362	0.272
Amenity grass	59.8	0.059	**-0.771**
Meadow	41.9	0.043	**0.646**
Trees	8.4	-0.088	-0.277

After varimax raw rotation, highly significant loading factors of the variables on the PCA axes are emboldened. Comm. (%) is the percentage of the total communality of each variable extracted by the first two PCA axes.

A generalized linear mixed model (GLMM) with Gaussian error and identity link function was carried out on the summary variables (bird species richness, total number of birds, Gini-Simpson Index of Diversity) from all 156 surveyed squares and both months. Two variables: environment type (urban/rural) and month (December/January) and their interaction were fixed categorical factors. Number of bird feeders, human population size, longitude, latitude, PCA1 and PCA2 scores (described above) were covariates. Interaction terms between environment type and covariates were also included in GLMMs to test for a different response of dependent variables to covariates in the two environments.

Observer identity, urban area pairing and square identity were random blocking factors in the GLMMs. Square identity was nested in urban-rural area pair. We used Akaike Information Criterion (AICc) to select the best reduced model and we present results for models which had values of ΔAICc (the difference between the models with lowest AICc and the given model) below 2 [34]. We used model averaging to get estimates of the function slopes (using a 99% confidence set).

Similar GLMMs were built for 10 of the most abundant species to test if environment type, geographical location, PCA1 and PCA2 scores, number of bird feeders and human population size affected their abundance. Interaction terms between the environment type and covariates were also included. Random factors were the same as described above. When analysing these species we encountered right-skewed distributions which are typical for count data with zeros. Thus, for species with excess zero counts we fitted a GLMM with a negative binomial error and logarithmic link function. The choice of Gaussian or negative binomial error variance was determined by examining AICc scores and the model with the lowest AICc was chosen [34].

Moreover, we also built GLMMs for all individual species with reasonable sample size, testing differences in abundance between the two environments and between the two months. The effect of covariates was omitted from this analysis. Bird species with low counts (< 10 individuals) were not individually analysed. The choice of Gaussian or negative binomial error variance was determined by examining AICc scores as described above.

Unless otherwise stated all GLMMs and correlation analyses were carried out using the SPSS 21 package [35]. An ordination of the mean counts for the 26 urban and 26 rural areas (i.e. averaged across three squares and two months) was undertaken in the CANOCO package [36]. We used a Detrended Correspondence Analysis (DCA), a multivariate statistical technique widely used by ecologists, to elucidate the relationships between biological assemblages of species and their environment [36, 37]. We used DCA because most ordination methods suffer from two major problems: the arch effect (caused by unimodal species response curves) and compression of the ends of the environmental gradient. Because of the first problem, the second ordination axis is an artefact and cannot be interpreted. The second problem is that the spacing of species (or samples) along the first axis is not necessarily related to the amount of change along the primary gradient. DCA overcomes these problems by dividing the first axis into segments, and rescales each segment to have mean value of zero on the second axis—this effectively compresses the curve to become flat. It also rescales the axis so that the ends are no longer compressed relative to the middle [37]. Species data were *log* x+1 transformed and downweighted for rare species in DCA. Downweighting was applied because ordination analyses are sensitive to rare species which influence analytical results to a much greater extent than would be predicted by their abundance [36]. The downweighting procedure replaces the abundance values of rare species in the data set, *a*ij, with new values, *a*ij’. A species is defined as being rare if its frequency in the data set, *f*1, is lower than *f*i,max/5, where *f*i,max is the maximum frequency of any species. For the rare species, the formula [38] for downweighted abundance is: *a*ij’ = *a*ij × [*f*i/(*f*i,max/5)]. DCA was carried out with the above mentioned twelve environmental variables (seven cover variables, environment type, bird feeders, human population size, latitude and longitude) used as supplementary variables, i.e. not influencing the original ordination [36].

In our analyses we performed multiple tests. However, we did not apply corrections for multiple testing. There are two basic reasons for this decision. First, we tested hypotheses on different species and obviously each species has a unique life-history and different biology. Therefore, there is no reason to assume that species responses would behave as random statistical processes. Secondly, the number of species tested was high, thus if the correction for multiple tests had been applied then one would not have been able to effectively test any hypothesis (for example with 50 species tested the Bonferroni corrected critical p value is 0.001 which means that tests are unfeasible and interpretation impossible). This problem has been discussed in many papers and the pitfalls of using such corrections are discussed in a paper by Garcia [39]. However, we provide information in the text and tables about corrected critical p-values for each set of tested hypotheses after using the Benjamini-Hochberg method for false discovery rates in multiple statistical tests [40].

Means are given with standard errors (SE).

## Results

**Fig 2:**
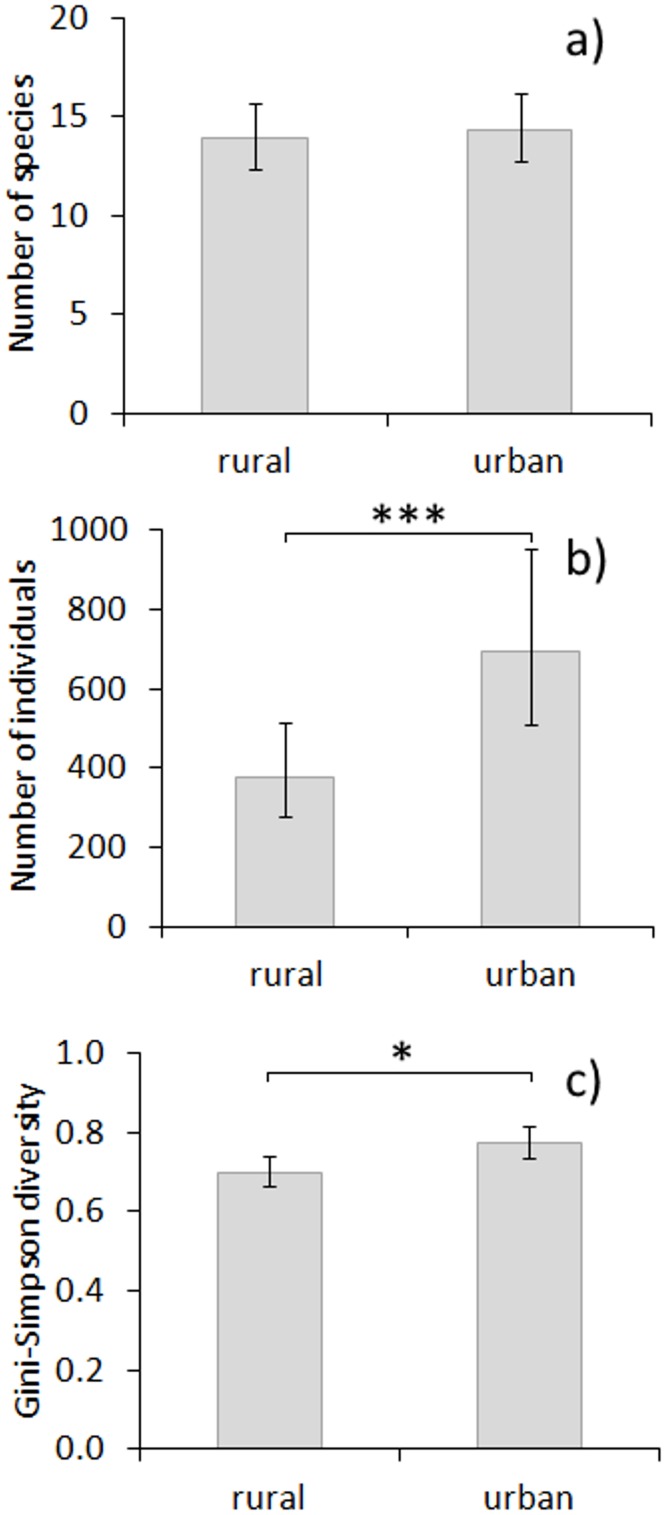
The effect of environment type on wintering birds. The effect of environment type on mean (a) species richness, (b) abundance and (c) species diversity of wintering birds. Whiskers are 95% confidence intervals.

**Fig 3:**
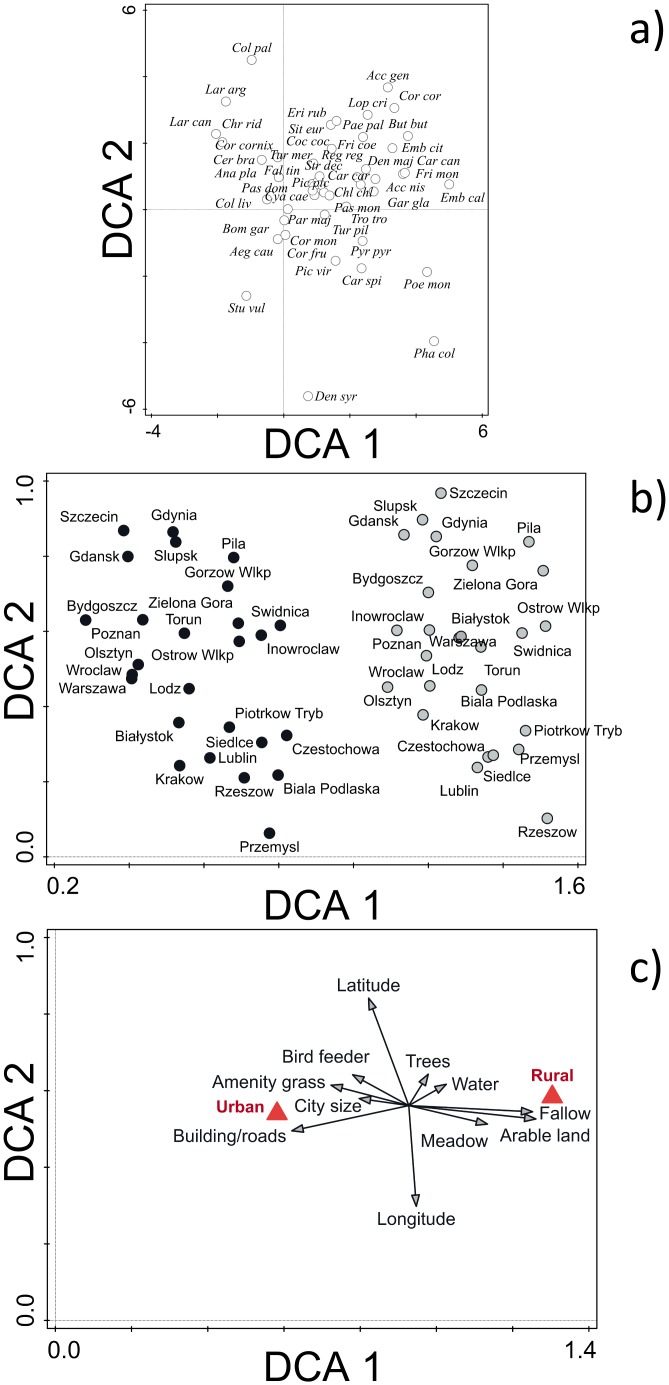
The DCA with environmental variables carried out on bird count data. The DCA with supplementary environmental variables carried out on the bird count data from Polish urban areas and paired rural areas. A. Species codes (Table 5) are shown for the 48 most common species; the remaining codes omitted and some jittering of codes has been done for clarity, B. The ordination of locations (grey symbol = rural, solid black symbol = urban), C. The ordination of supplementary environmental variables.

A total of 72 bird species and 89,710 individual birds were recorded in this study. Across all sites, nine species were only recorded as singletons, at the other extreme there were 18,864 records of House Sparrow *Passer domesticus*. The best model explaining species richness contained the effect of month (Tables 2 and 3). Mean species richness was lower in December than in January (Table 3). The number of species did not differ between the two environments (Fig 2). Mean species richness in the urban environment was 14.18±0.86 in December and 15.86±0.86 in January. In the rural environment species richness was 14.25±0.86 in December and 16.50±0.86 in January. The best model explaining abundance of birds included two variables: environment type and month. Abundance in urban areas was higher than in rural areas (Tables 2 and 3, Fig 2) and was higher in January than December (Tables 2 and 3, Fig 3). Mean abundance in the urban environment was 625.9±1.2 in December and 774.3±1.2 in January. In the rural environment mean abundance was 307.1±1.2 in December and 466.4±1.2 in January. There were two best models explaining species diversity (Table 2). Species diversity in urban areas was higher than in rural areas (Tables 2 and 3, Fig 2). Mean species diversity index in the urban environment was 0.76±0.02 both in December and January. In rural environment the index was 0.73±0.02 in December and 0.74±0.02 in January. Species diversity also increased with PCA1 (increasing proportion of open agricultural areas) but this effect was stronger in rural areas (Tables 2 and 3, significant interaction between environment type and PCA1). We found that the observer effect was non-significant in all analyses (S3 Table). City identity did not contribute in a significant way to variation in bird abundance, richness or diversity index (S3 Table). Among random effects only square identity was always significant which is trivial since squares differed in habitat composition from each other. Full sets of tested GLMMs for species richness, abundance and species diversity index are presented in S4, S5 and S6 Tables, respectively.

**Table 2:** Best generalized linear mixed models (GLMM) describing species richness, abundance and species diversity of birds in rural and urban areas during winter.

Models	AICc	-2log	ΔAICc	*w*
*SPECIES RICHNESS (corrected)*				
1. Month	219.386	211.255	0	0.934
*ABUNDANCE (corrected)*				
1. Environment+Month	766.861	758.73	0	0.689
*SPECIES DIVERSITY (corrected)*				
1. PCA1	-520.031	-528.162	0	0.246
2. Environment+PCA1+Environment×PCA1	-519.491	-527.623	0.54	0.188

The Akaike information criterion score (AICc), the -2log, the difference between the given model and the most parsimonious model (Δ) and the Akaike weight (*w*) are listed. Explanation of variable codes: Month—month of survey (December vs. January), Environment—environment type (rural vs. urban), PCA1—the first principal component of environmental variables describing the gradient of increasing proportion of open agricultural habitats.

**Table 3:** Averaged estimates of the function slopes of variables present in the most parsimonious GLMMs describing the corrected species richness, abundance and species diversity of birds in rural and urban areas during winter.

Variable	Estimate	SE	Lower 95% CL	Upper 95% CL	F(df1, df2)	P
***SPECIES RICHNESS (corrected)***						
**Month**					22.02 (1, 155)	<0.001
Month = December	-0.130	0.028	-0.185	-0.075		
Month = January	0*					
***ABUNDANCE (corrected)***						
**Environment**					31.49 (1, 129)	<0.001
Environment = Rural	-0.609	0.109	-0.824	-0.395		
Environment = Urban	0*					
**Month**					22.32 (1, 155)	<0.001
Month = December	-0.316	0.067	-0.448	-0.395		
Month = January	0*					
***SPECIES DIVERSITY (corrected)***						
**Environment**					5.08 (1, 178)	0.025
Environment = Rural	-0.026	0.011	-0.048	-0.004		
Environment = Urban	0*					
**PCA1**	0.012	0.004	0.005	0.020	11.40 (1, 190)	0.001
Environment**×PCA1**					12.34 (1, 213)	0.001
PCA1 in Rural	0.026	0.007	0.011	0.040		
PCA1 in Urban	0*					

Standard errors (SE) and 95% confidence limits (CL) are also presented. Tests of significance of variables are given in the final two columns.

* A reference variable

The mean difference between the uncorrected and corrected number of species was higher in rural areas (mean difference 2.213±0.253) than in urban areas (mean difference 1.395±0.253, GLMM F1,286 = 6.79, P = 0.010), indicating that species detectability was probably slightly lower in rural environments after accounting for habitat, month and all random effects. The estimates of detectability for individual species are presented in S7 Table.

The first two axes of the DCA explained 25.5% and 11.0% respectively (sum 36.5%) of the variance in the bird count data (Fig 3). The supplementary environmental variables explained 51.2% of the variance in the species-environment relationship. Attributes associated with rural areas were grouped to the right of axis 1 and those of urban areas to the left of this axis. Axis 2 appears to be a geographical (mainly longitudinal) gradient. There was almost a complete separation of rural and urban bird communities on axis 1 (Fig 3).

GLMMs were built for 49 individual species with abundances greater than 10 individuals (Table 4). These models revealed 27 species had a statistically significant preference; 17 to rural areas and 10 to urban areas (Table 4). For example 100% of Common gulls *Larus canus* were recorded in urban areas whilst 95% of 593 Yellowhammers *Emberiza citrinella* were recorded in rural areas (Table 4). The most widespread species was Great Tit *Parus major*, absent from just two of the 312 square/month combinations.

**Table 4:** The percentage of the 156 square/month combinations for both rural (R) and urban (U) areas in which each species (at least one individual) was recorded, the total number of individuals (n) recorded, the mean number for rural and urban areas, the percentage of records recorded from urban areas (%U), whether the model was based on negative binomial (N) or Gaussian (G) distribution, and the significance level of rural/urban, month and interaction terms from GLMM (month means not shown to save space).

Species	code	% presence	% presence	n	mean per survey	mean per survey	%U	Model	P	P	P
		R	U		R	U			R *vs* U(BH = 0.021)	Month(BH = 0.004)	Interaction(BH = none)
Goshawk *Accipiter gentilis* (Linnaeus 1758)	Acc gen	4	2	12	0.06	0.02	25	N	0.318	0.381	0.349
Eurasian Sparrowhawk *Accipiter nisus* (Linnaeus 1758)	Acc nis	17	6	38	**0.19**	0.06	24	G	0.003	0.087	0.492
Long-tailed Tit *Aegithalos caudatus* (Linnaeus 1758)	Aeg cau	3	3	48	0.08	**0.22**	73	N	<0.001	0.017	0.716
Mallard *Anas platyrhynchos* (Linnaeus 1758)	Ana pla	7	8	1119	0.72	6.45	90	N	0.142	0.830	0.684
Grey Heron *Ardea cinerea* (Linnaeus, 1758)	Ard cin	1	0	2	0.01	0.00	0		-	-	-
Long-eared Owl Asio *otus* (Linnaeus 1758)	Asi otu	0	1	1	0.00	0.01	100		-	-	-
Bohemian Waxwing *Bombycilla garrulus* (Linnaeus 1758)	Bom gar	13	20	2336	3.69	11.28	75	N	0.281	0.787	0.850
Common Buzzard *Buteo buteo* (Linnaeus 1758)	But but	11	1	23	**0.13**	0.01	9	G	<0.001	0.006	0.041
Rough-legged Buzzard *Buteo lagopus* (Pontoppidan 1763)	But lag	1	1	2	0.01	0.01	50		-	-	-
Common Linnet *Carduelis cannabina* (Linnaeus 1758)	Car can	6	2	112	0.69	0.03	4	G	0.121	0.180	0.212
European Goldfinch *Carduelis carduelis* (Linnaeus 1758)	Car car	13	5	183	1.03	0.15	13	N	0.111	0.625	0.431
Common Redpoll *Carduelis flammea* (Linnaeus 1758)	Car fla	1	1	11	0.01	0.06	91	N	0.575	0.087	0.395
Twite *Carduelis flavirostris* (Linnaeus 1758)	Car fla	3	1	26	0.13	0.04	23	G	0.194	0.016	0.194
Eurasian Siskin *Carduelis spinus* (Linnaeus 1758)	Car spi	23	12	998	**5.51**	0.88	14	N	0.035	0.566	0.348
Short-toed Treecreeper *Certhia brachydactyla* (Brehm 1820)	Cer bra	2	6	19	0.03	0.10	79	G	0.057	0.862	0.602
Eurasian Treecreeper *Certia familiaris* (Linnaeus 1758)	Cer fam	1	0	1	0.01	0.00	0		-	-	-
European Greenfinch *Chloris chloris* (Linnaeus 1758)	Chl chl	65	64	3152	9.28	10.93	54	N	0.820	<0.001	0.926
Black-headed Gull *Chroicocephalus ridibundus* (Linnaeus 1766)	Chr rid	3	38	1674	0.04	**10.69**	100	N	<0.001	0.553	0.553
Hawfinch *Coccothraustes coccothraustes* (Linnaeus 1758)	Coc coc	34	40	269	0.78	0.94	55	N	0.523	0.038	0.476
Feral Pigeon *Columba livia domestica* (Gmelin 1789)	Col liv	17	87	16648	4.58	**102.15**	96	N	<0.001	0.273	0.334
Common Wood Pigeon *Columba palumbus* (Linnaeus 1758)	Col pal	3	13	296	0.13	1.76	93	N	0.342	0.440	0.377
Common Raven *Corvus corax* (Linnaeus 1758)	Col cor	12	1	34	**0.21**	0.01	3		0.001	0.076	0.155
Hooded Crow *Corvus cornix* (Linnaeus 1758)	Cor cornix	17	33	310	0.47	**1.51**	76	N	0.025	0.727	0.906
Carrion Crow Corvus *corone* (Linnaeus 1758)	Cor coro	0	1	1	0.00	0.01	100	-	-	-	-
Rook *Corvus frugilegus* (Linnaeus 1758)	Cor fru	41	96	10597	12.13	**55.80**	82	N	<0.001	0.223	0.795
Jackdaw *Corvus monedula* (Linnaeus 1758)	Cor mon	39	96	7921	4.97	**45.81**	90	N	<0.001	0.217	0.706
Eurasian Blue Tit *Cyanistes caeruleus* (Linnaeus 1758)	Cya cae	85	92	1631	4.85	5.61	54	N	0.171	0.028	0.616
Great Spotted Woodpecker *Dendrocopos major* (Linnaeus 1758)	Den maj	32	14	110	**0.54**	0.16	23	G	0.006	0.616	0.568
Middle Spotted Woodpecker *Dendrocopos medius* (Linnaeus 1758)	Den med	4	1	9	0.05	0.01	11		-	-	-
Lesser Spotted Woodpecker *Dendrocopos minor* (Linnaeus 1758)	Den min	2	0	3	0.02	0.00	0		-	-	-
Syrian Woodpecker *Dendrocopos syriacus* (Hemprich & Ehrenberg, 1833)	Den syr	3	5	16	0.03	0.07	69	N	0.211	0.181	0.372
Black Woodpecker *Dryocopus martius* (Linnaeus 1758)	Dry mar	1	0	1	0.01	0.00	0		-	-	-
Corn Bunting *Emberiza calandra* (Linnaeus 1758)	Emb cal	4	0	24	0.15	0.00	0		-	-	-
Yellohammer *Emberiza citrinella* (Linnaeus 1758)	Emb cit	39	3	593	**3.60**	0.21	5	N	0.042	<0.001	0.506
Common Reed Bunting *Emberiza schoeniclus* (Linnaeus 1758)	Emb sch	2	0	4	0.03	0.00	0		-	-	-
European Robin *Erithacus rubecula* (Linnaeus 1758)	Eri rub	23	15	100	**0.46**	0.19	29	G	0.004	0.220	0.585
Common Kestrel *Falco tinnunculus* (Linnaeus 1758)	Fal tin	3	5	13	0.03	0.05	62	N	0.415	0.034	0.362
Common Chaffinch *Fringilla coelebs* (Linnaeus 1758)	Fri coe	31	25	274	0.95	0.81	46	N	0.747	0.014	0.877
Brambling *Fringilla montifringilla* (Linnaeus 1758)	Fri mon	4	1	17	**0.10**	0.01	6	N	0.025	0.170	0.286
Crested Lark *Galerida cristata* (Linnaeus 1758)	Gal cri	3	1	14	0.06	0.03	29		-	-	-
Eurasian Jay *Garrulus glandarius* (Linnaeus 1758)	Gar gla	51	14	297	**1.59**	0.31	16	G	<0.001	0.557	0.557
White-tailed Eagle *Haliaeetus albicilla* (Linnaeus 1758)	Hal alb	0	1	1	0.00	0.01	100		-	-	-
Great Grey Shrike *Lanius excubitor* (Linnaeus 1758)	Lan exc	1	0	1	0.01	0.00	0		-	-	-
Herring Gull *Larus argentatus* (Pontoppidan 1763)	Lar arg	1	23	305	0.02	**1.94**	99	G	<0.001	0.018	0.022
Common Gull *Larus canus* (Linnaeus 1758)	Lar can	0	26	810	0.00	**5.19**	100	G	<0.001	0.673	0.673
European Crested Tit *Lophophanes cristatus* (Linnaeus 1758)	Lop cri	9	3	35	0.18	0.04	20	G	0.072	0.492	0.378
Red Crossbill *Loxia curvirostra* (Linnaeus 1758)	Lox cur	1	1	7	0.03	0.01	29		-	-	-
Willow Tit *Poecile montanus* (Linnaeus 1758)	Poe mon	6	0	13	**0.08**	0.00	0	G	0.010	0.765	0.765
Marsh Tit *Poecile palustris* (Linnaeus 1758)	Poe pal	15	4	64	**0.37**	0.04	11	G	<0.001	0.726	0.599
Great Tit *Parus major* (Linnaeus 1758)	Par maj	99	100	8131	22.02	**30.11**	58	G	<0.001	0.795	0.379
House Sparrow *Passer domesticus* (Linnaeus 1758)	Pas dom	87	96	18864	52.75	68.17	56	G	0.089	<0.001	0.786
Eurasian Tree Sparrow *Passer montanus* (Linnaeus 1758)	Pas mon	69	55	3914	**17.32**	7.77	31	G	0.003	0.589	0.245
Coal Tit *Periparus ater* (Linnaeus 1758)	Per ate	24	3	110	**0.62**	0.08	12	G	0.002	0.174	0.022
Grey Partridge *Perdix perdix* (Linnaeus 1758)	Per per	1	0	11	0.07	0.00	0		-	-	-
Great Cormorant *Phalacrocorax carbo* (Linnaeus 1758)	Pha car	0	1	1	0.00	0.01	100		-	-	-
Common Pheasant *Phasianus colchicus* (Linnaeus 1758)	Pha col	8	0	25	**0.16**	0.00	0	G	0.002	0.890	0.890
Black Redstart *Phoenicuros ochruros* (Gmelin 1774)	Pho och	0	1	1	0.00	0.01	100		-	-	-
Magpie *Pica pica* (Linnaeus 1758)	Pic pic	90	81	2226	5.85	**8.42**	59	G	0.016	0.464	0.582
European Green Woodpecker *Picus viridis* (Linnaeus 1758)	Pic vir	2	1	5	0.02	0.01	40		-	-	-
Dunnock *Prunella modularis* (Linnaeus 1758)	Pru mod	2	0	3	0.02	0.00	0		-	-	-
Eurasian Bullfinch *Pyrrhula pyrrhula* (Linnaeus 1758)	Pyr pyr	27	13	229	0.98	0.49	33	G	0.419	0.502	0.491
Goldcrest *Regulus regulus* (Linnaeus 1758)	Reg reg	20	5	88	**0.45**	0.12	20		0.003	0.023	0.087
Eurasian Serin *Serinus serinus* (Linnaeus 1766)	Ser ser	1	1	6	0.01	0.03	67	G	-	-	-
Eurasian Nuthatch *Sitta europea* (Linnaeus 1758)	Sit eur	15	7	66	0.28	0.14	33	G	0.143	0.860	0.380
Eurasian Collared Dove *Streptopelia decaocto* (Frivaldszky 1838)	Str dec	56	78	2633	**8.61**	8.27	49	N	0.031	0.666	0.870
Common Starling *Sturnus vulgaris* (Linnaeus 1768)	Stu vul	1	6	103	0.01	0.65	98	G	0.074	0.165	0.188
Eurasian Wren *Troglodytes troglodytes* (Linnaeus 1758)	Tro tro	6	2	19	**0.10**	0.03	21	G	0.038	0.015	0.015
Redwing *Turdus iliacus* (Linnaeus 1766)	Tur ili	2	1	9	0.02	0.04	67		-	-	-
Common Blackbird *Turdus merula* (Linnaeus 1758)	Tur mer	81	75	1959	5.78	6.78	54	N	0.420	0.001	0.305
Song Thrush *Turdus philomelos* (Brehm 1831)	Tur phi	0	1	1	0.00	0.01	100		-	-	-
Fieldfare *Turdus pilaris* (Linnaeus 1758)	Tur pil	60	58	1128	4.24	2.99	41	N	0.193	0.677	0.028
Mistle Thrush *Turdus viscivorus* (Linnaeus 1758)	Tur vis	1	1	3	0.01	0.01	33		-	-	-

Benjamini-Hochberg corrected significance level (BH) is given in brackets under a header of the columns for each hypothesis. Codes are used in Fig 2A. Where rural/urban comparisons were significantly different the higher mean is in bold. Species in alphabetical order of Latin names.

GLMMs testing the effect of environmental variables on the abundance of the ten most numerous species are shown in Tables 5 and 6. Results were mixed, but negative effects of longitude and land use intensity (PCA1) were apparent for several species. Interestingly, in the best models there were statistically significant interactions between environment type and geographical variables (Tables 5 and 6). The best model explaining abundance of House Sparrow contained the effects of month, human population size and latitude (Table 5). The abundance of this species decreased with latitude but increased with human population size (Table 6). Abundance of this species was also lower in December than in January (Table 6).

**Table 5:** Best generalized linear mixed models (GLMM) describing the abundance of the 10 most numerous bird species during the winter.

Species and models	AICc	-2log	Δ AICc	*w*
**House Sparrow *Passer domesticus***				
CitySize+Latitude	937.194	931.115	0	0.548
Latitude+Month	939.187	933.509	1.993	0.202
**Feral Pigeon *Columba livia***				
Environment+Environment×CitySize	120.222	118.903	0	0.543
CitySize	120.754	119.214	0.532	0.416
Environment+CitySize	122.005	120.621	1.783	0.223
**Rook *Corvus frugilegus***				
CitySize+Longitude	206.931	200.850	0	0.469
Longitude+Environment	207.134	201.090	0.203	0.424
Environment+Environment×Longitude+Environment×PCA1	207.681	201.420	0.75	0.322
CitySize+Longitude+Environment+Environment×PCA1	207.905	201.460	0.974	0.288
CitySize+Environment×PCA1	208.432	203.245	1.501	0.221
Environment+Environment×Longitude	208.753	203.530	1.822	0.189
**Great Tit *Parus major***				
Environment+PCA1	572.933	564.800	0	0.231
PCA1	574.621	567.223	1.688	0.100
**Jackdaw *Corvus monedula***				
Environment	192.134	186.051	0	0.474
Environment+CitySize+Environment×Longitude	193.045	186.433	0.911	0.301
Environment×Longitude	193.953	187.832	1.819	0.191
Environment+Environment×Longitude	194.106	187.653	1.972	0.177
**Eurasian Greenfinch *Chloris chloris***				
Month	942.729	936.735	0	0.166
Month+Feeders	942.944	936.366	0.215	0.149
Month+Environment×Feeders	943.953	937.004	1.224	0.090
Month+Environment×CitySize	944.305	937.970	1.576	0.075
Month+Feeders+Environment×CitySize	944.588	938.511	1.859	0.065
**Eurasian Tree Sparrow *Passer montanus***				
Environment	430.531	422.620	0	0.435
**Eurasian Collared Dove *Streptopelia decaocto***				
Environment+Feeders+PCA2+CitySize	331.590	325.352	0	0.393
Environment+Feeders+CitySize	331.770	326.843	0.18	0.359
Environment+PCA1, CitySize	332.180	326.920	0.59	0.293
Environment+PCA1+CitySize+Environment×PCA1	333.000	327.640	1.41	0.194
Environment+Feeders+CitySize+Environment×PCA1	333.430	327.781	1.84	0.157
**Bohemian Waxwing *Bombycilla garrulus***				
Longitude	842.283	838.806	0	0.135
Longitude+PCA1	843.000	839.051	0.717	0.094
Environment +Longitude	844.103	839.728	1.82	0.054
Environment + Environment×Longitude	844.218	839.815	1.935	0.051
Longitude+ Environment +PCA2	844.280	840.269	1.997	0.050
**Magpie *Pica pica***				
Environment+PCA1	453.732	446.214	0	0.109
CitySize	454.205	446.837	0.473	0.086
CitySize+PCA1	454.687	446.910	0.955	0.067
Environment+Environment×PCA1	454.958	447.042	1.226	0.059
CitySize+Environment×PCA1	455.178	447.522	1.446	0.053
Environment+Environment×CitySize	455.629	447.448	1.897	0.042

The Akaike information criterion score (AICc), the -2log, difference between the given model and the most parsimonious model (Δ) and the Akaike weight (*w*) are listed. Explanation of variable codes: Feeders—number of bird feeders, CitySize—human population size in the city, Month—month of survey (December vs. January), Environment—type of the environment (urban vs. rural), Longitude—geographical longitude, PCA1—the first principal component of environmental variables describing the gradient of increasing proportion of open agricultural habitats, PCA2—the second principal component of environmental variables describing gradient from semi-natural grasslands to intensively managed amenity grasses.

**Table 6:** Averaged estimates of the function slopes of variables present in the most parsimonious GLMMs describing the corrected abundance of the 10 most numerous recorded bird species.

Variable	Estimate	SE	Lower 95% CL	Upper 95% CL	F(df1, df2)	P
**House Sparrow *Passer domesticus***						
**City size**	0.257	0.250	-0.037	0.562	2.95 (1, 298)	0.087
**Latitude**	-0.370	0.131	-0.629	-0.114	8.06 (1, 297)	0.005
**Month**					8.01 (1, 298)	0.006
Month = December	-0.246	0.110	-0.462	-0.030		
Month = January	0*					
**Feral Pigeon *Columba livia***						
**Environment**					9.84 (1, 273)	0.002
Environment = Rural	-603.185	192.3	-981.7	-224.7		
Environment = Urban	0*					
**CitySize**	37.3	9.2	19.1	55.4	5.50 (1, 77)	0.022
**Environment×CitySize**					12.98 (1, 188)	<0.001
CitySize in Rural	40.5	11.2	18.2	62.7		
CitySize in Urban	0*					
**Rook *Corvus frugilegus***						
**CitySize**	0.559	0.206	0.154	0.964	17.48 (1, 300)	<0.001
**Environment**					4.09 (1, 301)	0.044
Environment = Rural	-0.337	0.167	-0.666	-0.009		
Environment = Urban	0*					
**Environment×PCA1**					15.29 (1, 301)	<0.001
PCA1 in Rural	-0.590	0.151	-0.887	-0.293		
PCA1 in Urban	0*					
**Longitude**	-0.052	0.025	-0.101	-0.005	5.31 (1, 299)	0.023
**Longitude×Environment**					4.32 (1, 301)	0.038
Longitude in Rural	-0.234	0.080	-0.391	-0.090		
Longitude in Urban	0*					
**Great Tit *Parus major***						
**Environment**					3.87 (1, 203)	0.049
Environment = Rural	-9.134	4.630	-18.209	-0.059		
Environment = Urban	0*					
**PCA1**	1.654	0.723	0.228	3.080	5.24 (1, 190)	0.023
**Jackdaw *Corvus monedula***						
**Environment**					4.09 (1, 295)	0.044
Environment = Rural	-23.9	9.3	4.5	42.3		
Environment = Urban	0*					
**CitySize**	0.219	0.106	0.004	0.453	4.10 (1, 294)	0.042
**Environment×Longitude**					5.37 (1, 294)	0.021
Longitude in Rural	-0.221	0.095	-0.409	-0.033		
Longitude in Urban	0*					
**European Greenfinch *Chloris chloris***						
**Month**					18.44 (1, 169)	<0.001
Month = December	-9.593	2.625	-14.772	-4.414		
Month = January	0*					
**Feeders**	1.273	0.603	0.091	2.455	4.00 (1, 204)	0.045
**Environment×Feeders**					3.99 (1, 250)	0.047
Feeders in Urban	4.450	1.725	1.051	7.849		
Feeders in Rural	0*					
**Environment×CitySize**					4.26 (1, 136)	0.041
CitySize in Rural	4.538	2.199	0.190	8.885		
CitySize in Urban	0*					
**Eurasian Tree Sparrow *Passer montanus***						
**Environment**					10.02 (1, 308)	0.002
Environment = Rural	4.775	1.509	1.807	7.745		
Environment = Urban	0*					
**Eurasian collared dove *Streptopelia decaocto***						
**Environment**					4.10 (1, 305)	0.044
Environment = Rural	0.183	0.089	0.009	0.357		
Environment = Urban	0*					
**Feeders**	0.118	0.062	-0.004	0.240	4.22 (1, 300)	0.069
**PCA2**	0.078	0.032	0.015	0.141	4.20 (1, 293)	0.018
**CitySize**	-0.298	0.119	-0.531	-0.065	4.17 (1, 285)	0.020
**PCA1**	-0.181	0.083	-0.344	-0.018	4.75 (1, 149)	0.030
**Environment×PCA1**					3.60 (1, 301)	0.057
PCA1 in Rural	-0.103	0.055	-0.211	0.005		
PCA1 in Urban	0*					
**Bohemian Waxwing *Bombycilla garrulus***						
**Longitude**	0.123	0.056	0.011	0.234	4.74 (1, 99)	0.030
**PCA1**	-0.271	0.136	-0.544	0.002	3.82 (1, 108)	0.052
**PCA2**	-0.282	0.131	-0.542	-0.024	4.63 (1, 302)	0.032
**Environment**					3.61 (1, 130)	0.063
Environment = Rural	-0.347	0.179	-0.699	0.003		
Environment = Urban	0*					
**Environment×Longitude**						
Longitude in Rural	0.288	0.182	-0.069	0.645	3.53 (1, 280)	0.092
Longitude in Urban	0*					
**Magpie Pica pica**						
**Environment**					7.06 (1, 90)	0.008
Environment = Rural	-1.198	0.330	-2.085	-0.311		
Environment = Urban	0*					
**PCA1**	-0.975	0.330	-1.624	-0.325	8.71 (1, 300)	0.003
**CitySize**	2.940	0.491	1.974	3.906	35.84 (1, 302)	<0.001
**Environment×PCA1**					3.35 (1, 190)	0.068
PCA1 in Rural	0.605	0.330	-0.045	1.254		
PCA1 in Urban	0*					
**Environment×CitySize**					6.96 (1, 293)	0.009
CitySize in Rural	-1.297	0.491	-2.262	-0.330		
CitySize in Urban	0*					

Standard errors (SE) and 95% confidence limits (CL) are also presented. Tests of significance of variables are given in the final two columns. Explanation of variable codes: Table 5.

* A reference variable

The best model explaining the abundance of Feral Pigeon *Columba livia* contained environment type, human population size and the interaction between these variables (Table 5). The abundance of this species increased with human population size and was higher in the urban environment (Table 6). The interaction term also indicated that the abundance of Feral Pigeons was more strongly correlated with human population size in the rural environment (Table 6).

The best model explaining the abundance of Rook *Corvus frugilegus* contained environment type, human population size, geographical longitude and PCA1 score (Table 5). Moreover, these models contained interaction terms between environment type and both longitude and PCA1 score (Table 5). The abundance of this species was higher in the urban environment, and also increased with human population size and decreased with longitude (Table 6). The negative impact of longitude on abundance was greater in the rural environment (Table 6). The effect of PCA1 (the increasing cover of open agricultural areas) on Rook abundance was negative in the rural, but positive in the urban, environment (Table 6).

The best models describing the abundance of Great Tit contained environment type and PCA1 (Table 5). The abundance of this species was higher in urban environments and it increased with PCA1 scores (the increasing cover of agricultural habitats) (Table 6).

The best model explaining the abundance of Jackdaw *Corvus monedula* contained environment type, human population size and the interaction between environment type and longitude (Table 5). The abundance of this species increased with human population size and was higher in the urban environment (Table 6). The abundance of Jackdaws decreased with longitude in rural, but not in urban, environments (Table 6).

The best models explaining the abundance of Greenfinch *Chloris chloris* contained month, feeder numbers, and the interaction between environment type and both number of bird feeders and human population size (Table 5). The abundance of Greenfinch was lower in December than in January and increased with the number of bird feeders (Table 6). The effect of bird feeder number on abundance was modified by the environment type; bird feeders had a greater positive effect on the number of Greenfinches in urban than in rural environments (Table 6). Similarly, human population size positively affected the abundance of this species but the relationship was stronger in rural environments (Table 6).

The best model describing the abundance of Eurasian Tree Sparrow only contained the effect of environment (Table 5). This species was more abundant in rural environments (Table 6).

The best models explaining the abundance of Eurasian Collared Dove *Streptopelia decaocto* contained environment type, number of bird feeders, PCA1, PCA2, human population size and the interaction between environment type and PCA1 (Table 5). The abundance of this species was higher in rural environments (Table 6). Abundance was positively correlated with the number of bird feeders and PCA2 scores (increasing cover of amenity grasses) but negatively with human population size and PCA1 scores (increasing cover of open agricultural habitats, Table 6). However, the effect of PCA1 was modified by the environment type; PCA1 had a positive effect on abundance in the urban, but not in the rural, environment (Table 6).

The best models explaining the abundance of Bohemian Waxwing *Bombycilla garrulus* contained environment type, longitude, PCA1 scores, PCA2 scores and the interaction between environment type and longitude (Table 5). The abundance of this species was higher in the urban environment and it increased with longitude but decreased with PCA1 (increasing cover of open agricultural habitats) and PCA2 (increasing cover of amenity grasses) (Table 6). However, the effect of longitude was modified by the environment type; the abundance increased with longitude in the rural, but not in the urban, environment (Table 6).

The best models describing the abundance of Magpie *Pica pica* contained environment type, human population size, PCA1 scores and two interaction terms: between environment type and both PCA1 and human population size (Table 5). The number of Magpies was higher in urban environments and increased with human population size but decreased with PCA1 scores (increasing cover of agricultural habitats) (Table 6). The effects of human population size and PCA1 were different in the two types of the environment. The positive effect of human population size was greater in urban environments and PCA1 had a stronger negative impact in urban environments (Table 6).

## Discussion

Our study shows differences between rural and urban areas in the number of individuals, the whole assemblage, as well as in the densities of particular species in winter. However, our results do not seem as well supported as those described in many studies during the breeding season [16,17,41]. Indeed, among our summary variables, we only detected statistical significance for the number of individuals, which, on average, was more than twice as high in urban than in rural areas. Species diversity was also higher in urban areas. However, for individual species there were strong preferences between rural and urban environments in winter, which is probably not related to urbanization *per se*, but to food availability, microhabitat preferences, and direct and indirect human activity [2,13,14].

Recently, many studies have indicated that rural and urban populations of birds differ from one another [8]). The main finding of our study, i.e. differences in the density of particular species, also supports this view. However, the factors affecting wintering bird communities were related not only to the main environment difference (urban *vs*. rural), but also to other variables. For example, our study clearly revealed that longitude, human population size and bird feeders have an important impact on wintering birds. The importance of these variables for birds, mostly during the breeding season, has been already identified (e.g. [2,8,9]). Areas located in western Poland had a significantly higher abundance of some species than those in the eastern part of the country. This is not surprising, because in western Poland the winter climate is characterized by higher temperature and lower snow cover [20]. Both these factors generally positively affect wintering bird species [12,16,42]. However, our results indicated that longitude had a stronger effect on some bird species in rural areas. For example, for Rooks and Jackdaws longitude negatively affected abundance, however the effect was greater in rural areas indicating that urban areas buffer against harsh winter climate mediated by geographical location. Thus, it is possible that urban environments located within colder areas are an especially good wintering habitat for birds and, consequently, urbanization processes may be especially rapid in towns and cities located in cold climates. The effect of human population size also positively influenced some birds, such as Eurasian Greenfinch and Magpie. A statistically significant interaction between this variable and environment type indicated that the positive effect of human population size was stronger in urban areas, suggesting dependence of bird populations on human-related resources in urban environment. The dependence of some species on human resources was also detected as a positive relationship between the number of bird feeders and bird abundance, e.g. in Eurasian Greenfinch or Eurasian Collared Dove.

The significant difference between early (December) and late (January) winter may be important in understanding changes in wintering bird communities. These changes are probably related to large geographical bird movements due to winter severity [23,43], because differences between environments are similar, as indicated by the non-significance of the interaction term in analyses. Results also indicate that birds in midwinter move closer to humans, both in cities and villages, because access to food is easier there, especially during snowy days [23,41,42].

Our results indicate that habitat variables are also important for the diversity of wintering species. To the best of our knowledge there are only a few large scale studies of birds wintering in rural and urban environments [44,45,46,47]. Studies in Finland [13,14] showed that residential areas had higher densities of birds during winter than areas occupied by other types of development, roads and open grassland, but generally those authors underlined the importance of cities to wintering birds under the harsh winter conditions in Finland. On the other hand, a negative effect of urban areas on the density and number of bird species in adjacent rural areas has been shown [48]. One potential explanation is that birds used urban areas for wintering and therefore avoided rural habitats in winter. For particular species, other traits of the study squares were also important, such as amenity grass (with a positive effect for some species), mainly used as a foraging place for birds, especially in bigger agglomerations [23]. Interestingly, our study suggests that urban areas may be important for many bird groups including seed-eating passerines and insectivores. Considering the strong decline of many common farmland birds in Europe, including sedentary species [49], it is of interest to note that not only rural habitats, including villages and small farms, but also urban areas may be one of the key habitats providing refuge and food resources, and, eventually may improve the winter survival of some farmland species [18,50].

As in every large-scale study our methodology has some issues that must be taken into account when interpreting results. Time spent on bird counting was long and pseudoreplication might have played a role. However, birds were noted on maps and carefully watched to avoid counting the same individuals more than once. Moreover, the generally low number of species allowed individual birds to be followed. On the other hand, if the duration of observation had been shorter, then problems in species detectability would have been more serious.

We found that detectability corrections played a role in analyses and interpretation of findings. The analysis of differences between uncorrected and corrected numbers of species revealed that observers usually detected, on average, one or two more species in the urban environment than in the rural one. It must also be stressed that for some species we were not able to calculate detectability due to their low numbers. This, however, should not affect inter-environment and inter-survey (December-January) analyses since these rarer species did not contribute much to total abundance.

In conclusion, we have shown that winter density and species diversity of birds differs between urban and rural areas, and that preferences for the two types of environment exist. Obviously those preferences appear to be highly species-specific, but in both environments birds are responding to environmental variables, such as habitat cover and geographic location (longitude) and human related food resources.

## Supporting Information

S1 Fig Correlation between the two methods for calculation of detectability. Correlation between the two methods for calculation of detectability. Whiskers are 95% confidence intervals calculated only for Royle’s estimator [30]. Spearman correlation coefficient is presented.
(TIF)

S2 Fig Correlation between bird body size and detectability. Correlation between bird body length and the estimator of detectability (Royle estimator[30]). Whiskers are 95% confidence intervals. Spearman correlation coefficient is presented.
(TIF)

S3 Fig Correlation between the observed number of bird species and estimated number of species. Correlation between the observed number of bird species and estimated number of species via bias-corrected Chao estimation [26, 27]for rural environment during December (a) and January (b), and for urban one (c, d). Spearman correlation coefficients are presented.
(TIF)

S4 Fig Correlation between the observed abundance of birds and estimated abundance. Correlation between the observed abundance of birds and estimated abundance via Royle’s correction [30] for rural environment during December (a) and January (b), and for urban environment (c, d). Spearman correlation coefficients are presented.
(TIF)

S5 Fig Principal component analysis on habitat cover variables. Ordination environmental variables describing cover of different habitat types along axes representing first two principal components (PCA).
(TIF)

S1 Table Location and details of the 26 urban environments (towns and cities); data extracted from www.wikipedia.org. Cities are arranged by human population size.
(DOC)

S2 Table Spearman correlation coefficients between environmental variables. Significant correlations are emboldened (significance level is in brackets).
(DOC)

S3 Table Estimation of random effects in GLMMs. (DOC)

S4 Table Generalized linear mixed models (GLMM) describing the species richness of birds in urban and rural areas during winter. The Akaike information criterion score (AICc), the -2log, difference between the given model and the most parsimonious model (Δ) and the Akaike weight (w) are listed. Explanation of variable codes: Month—month of survey (December vs. January), Environment—type of environment (urban vs. rural), Longitude—geographical longitude, Latitude—geographical latitude, PCA1—a first principal component of environmental variables describing the increasing cover of open agricultural habitats, PCA2—a second principal component of environmental variables describing the gradient from natural grasslands (meadows) to intensively managed amenity grassland, Feeders—number of bird feeders in a square plot, CitySize—human population size in the city. The best model is emboldened.
(DOC)

S5 Table Generalized linear mixed models (GLMM) describing the abundance of birds in urban and rural areas during winter. The Akaike information criterion score (AICc), the -2log, difference between the given model and the most parsimonious model (Δ) and the Akaike weight (w) are listed. Explanation of variable codes: Month—month of survey (December vs. January), Environment—type of environment (urban vs. rural), Longitude—geographical longitude, Latitude—geographical latitude, PCA1—a first principal component of environmental variables describing the increasing cover of open agricultural habitats, PCA2—a second principal component of environmental variables describing the gradient from natural grasslands (meadows) to intensively managed amenity grassland, Feeders—number of bird feeders in a square plot, CitySize—human population size in the city. The best model is emboldened.
(DOC)

S6 Table Generalized linear mixed models (GLMM) describing the Gini-Simpson bird diversity index in urban and rural areas during winter. The Akaike information criterion score (AICc), the -2log, difference between the given model and the most parsimonious model (Δ) and the Akaike weight (*w*) are listed. Explanation of variable codes: Month—month of survey (December vs. January), Environment—type of environment (urban vs. rural), Longitude—geographical longitude, Latitude—geographical latitude, PCA1—a first principal component of environmental variables describing the increasing cover of open agricultural habitats, PCA2—a second principal component of environmental variables describing the gradient from natural grasslands (meadows) to intensively managed amenity grassland, Feeders—number of bird feeders in a square plot, CitySize—human population size in the city. The best models are emboldened.
(DOC)

S7 Table Probability of detection for species in urban and rural landscapes derived from Kendall [31] and Royle [30] estimators. (DOC)
