# Supplementary figures and images for: Winter Bird Assemblages in Rural and Urban Environments: A National Survey
Source: PLoS One. 2015 Jun 18;10(6):e0130299. doi: 10.1371/journal.pone.0130299 (PMC4472663; doi:10.1371/journal.pone.0130299)

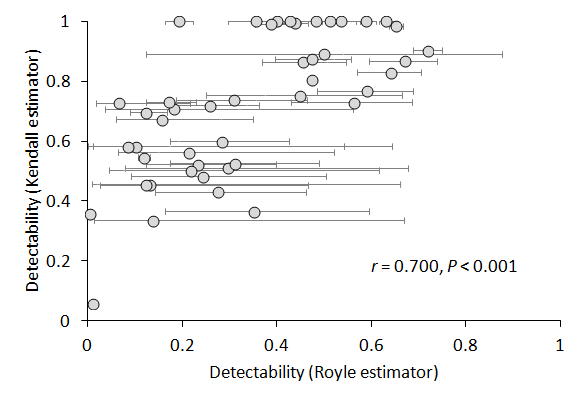

Supplement: S1 Fig — Correlation between the two methods for calculation of detectability. Whiskers are 95% confidence intervals calculated only for Royle’s estimator [30]. Spearman correlation coefficient is presented. (TIF) [file pone.0130299.s001.tif]

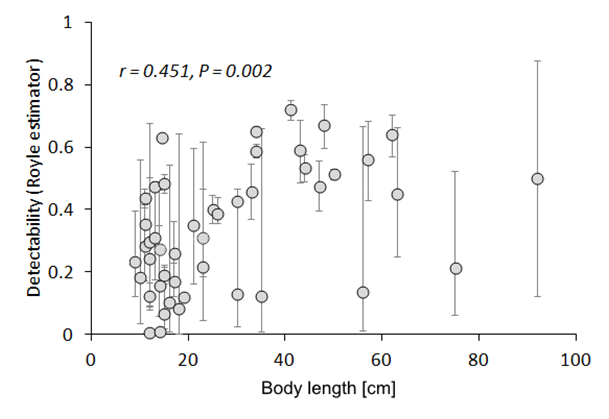

Supplement: S2 Fig — Correlation between bird body length and the estimator of detectability (Royle estimator[30]). Whiskers are 95% confidence intervals. Spearman correlation coefficient is presented. (TIF) [file pone.0130299.s002.tif]

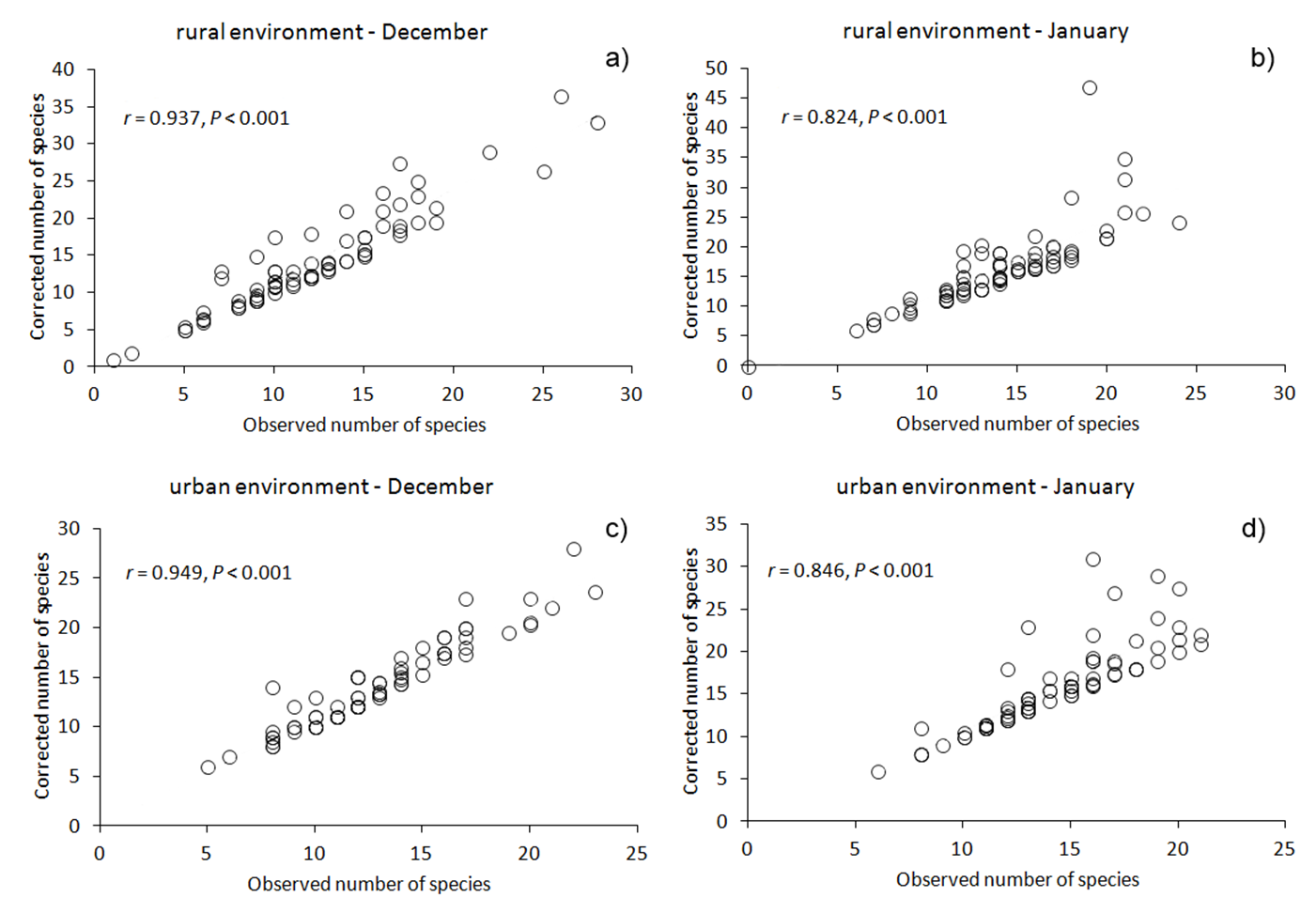

Supplement: S3 Fig — Correlation between the observed number of bird species and estimated number of species via bias-corrected Chao estimation [26, 27]for rural environment during December (a) and January (b), and for urban one (c, d). Spearman correlation coefficients are presented. (TIF) [file pone.0130299.s003.tif]

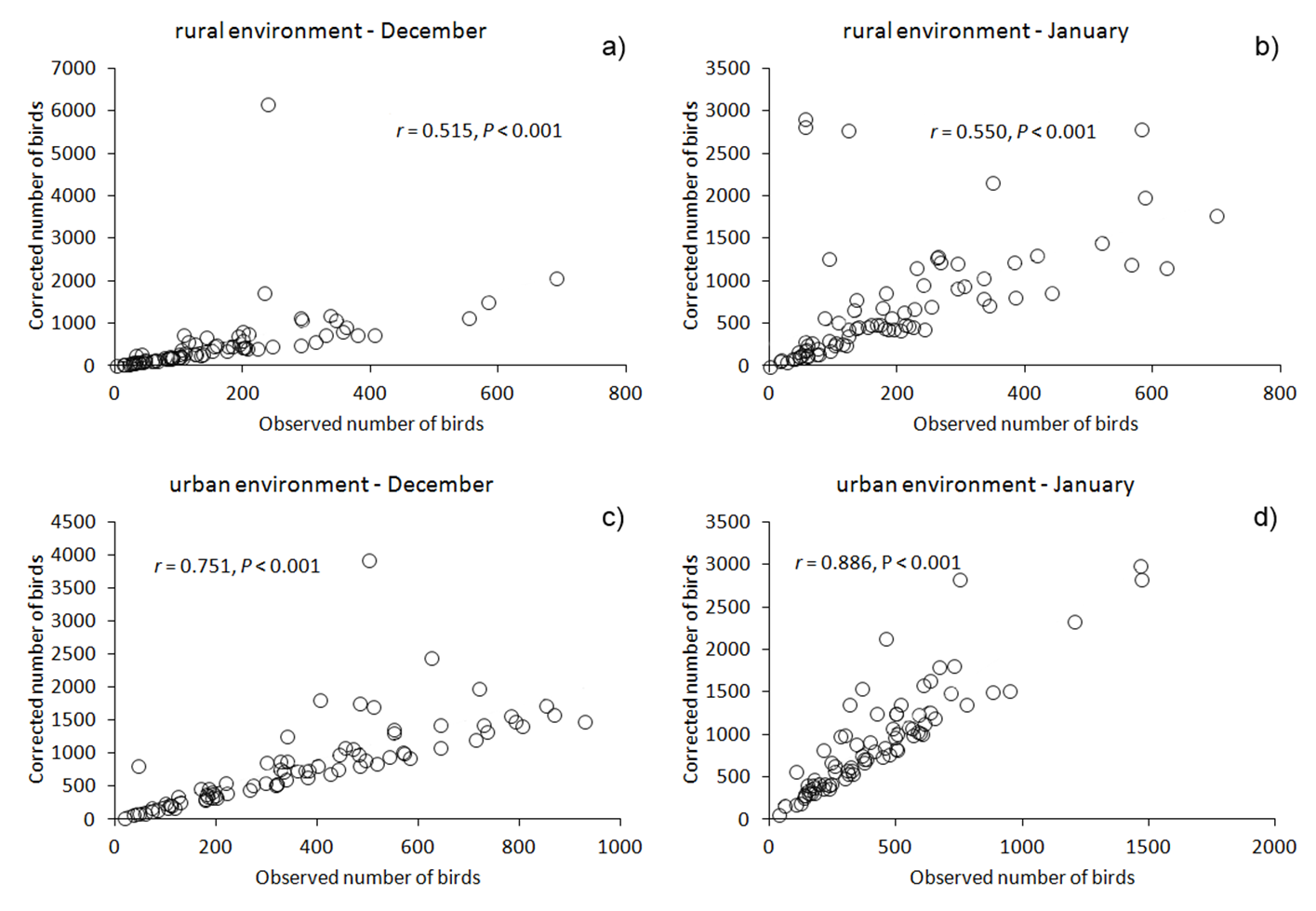

Supplement: S4 Fig — Correlation between the observed abundance of birds and estimated abundance via Royle’s correction [30] for rural environment during December (a) and January (b), and for urban environment (c, d). Spearman correlation coefficients are presented. (TIF) [file pone.0130299.s004.tif]

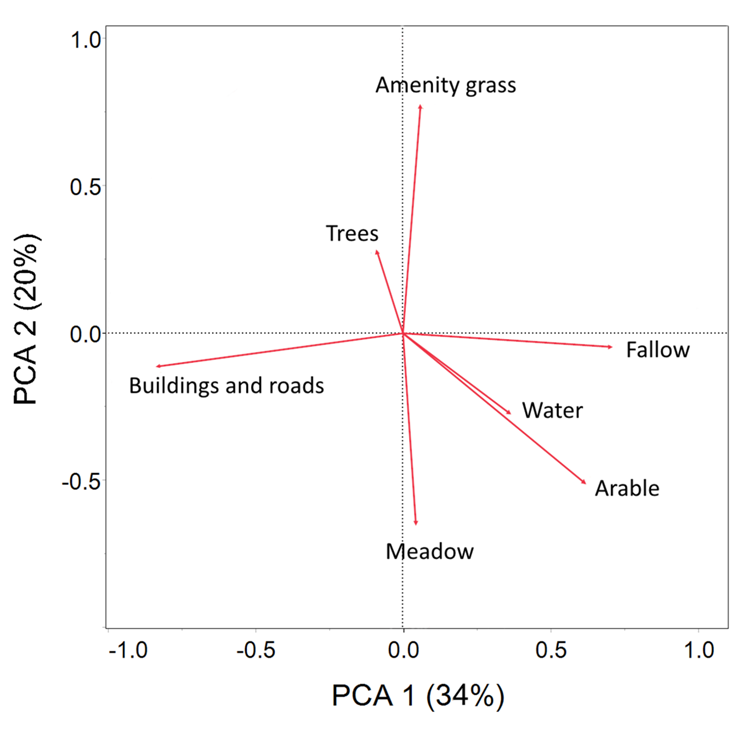

Supplement: S5 Fig — Ordination environmental variables describing cover of different habitat types along axes representing first two principal components (PCA). (TIF) [file pone.0130299.s005.tif]
