# Supplementary material for: Winter Bird Assemblages in Rural and Urban Environments: A National Survey
Source: PLoS One. 2015 Jun 18;10(6):e0130299. doi: 10.1371/journal.pone.0130299 (PMC4472663; doi:10.1371/journal.pone.0130299)
Supplement: S1 Table — Cities are arranged by human population size. (DOC) [file pone.0130299.s006.doc]

**S1 Table.** Location and details of the 26 urban environments (towns and cities); data extracted from www.wikipedia.org. Cities are arranged due to human population size.

| ID | City | Total population | Area (km^2^) | Latitude (N) | Longitude (E) |
| --- | --- | --- | --- | --- | --- |
| 1 | Warszawa | 1 711 324 | 517 | 52°13' | 21°0' |
| 2 | Kraków | 759 131 | 326 | 50°3' | 19°56' |
| 3 | Łódź | 725 055 | 293 | 51°45' | 19°27' |
| 4 | Wrocław | 631 377 | 293 | 51°6' | 17°2' |
| 5 | Poznań | 552 393 | 261 | 52°24' | 16°55' |
| 6 | Gdańsk | 460 517 | 261 | 54°21' | 18°38' |
| 7 | Szczecin | 409 211 | 300 | 53°25' | 14°33' |
| 8 | Bydgoszcz | 363 020 | 175 | 53°7' | 17°54' |
| 9 | Lublin | 348 567 | 147 | 51°14' | 22°34' |
| 10 | Białystok | 294 675 | 102 | 53°7' | 23°10' |
| 11 | Gdynia | 248 574 | 135 | 54°31' | 18°31' |
| 12 | Częstochowa | 235 798 | 170 | 50°49' | 19°7' |
| 13 | Toruń | 191 277 | 115 | 53°0' | 18°35' |
| 14 | Rzeszów | 180 031 | 116 | 50°2' | 21°59' |
| 15 | Olsztyn | 175 482 | 88 | 53°46' | 20°28' |
| 16 | Gorzów Wlkp. | 124 554 | 86 | 52°43' | 15°14' |
| 17 | Zielona Góra | 119 182 | 58 | 51°56' | 15°30' |
| 18 | Słupsk | 95 882 | 43 | 54°27' | 17°1' |
| 19 | Piotrków Tryb. | 76 717 | 67 | 51°24' | 19°42' |
| 20 | Siedlce | 76 480 | 31 | 52°10' | 22°17' |
| 21 | Inowrocław | 75 719 | 30 | 52°47' | 18°15' |
| 22 | Piła | 74 818 | 102 | 53°9' | 16°44' |
| 23 | Ostrów Wlkp. | 72 907 | 41 | 51°39' | 17°48' |
| 24 | Przemyśl | 64 728 | 46 | 49°47' | 22°46' |
| 25 | Świdnica | 60 213 | 21 | 50°50' | 16°29' |
| 26 | Biała Podlaska | 58 009 | 49 | 52°1' | 23°6' |
