# Supplementary material for: Winter Bird Assemblages in Rural and Urban Environments: A National Survey
Source: PLoS One. 2015 Jun 18;10(6):e0130299. doi: 10.1371/journal.pone.0130299 (PMC4472663; doi:10.1371/journal.pone.0130299)
Supplement: S2 Table — Significant correlations are emboldened (significance level is in brackets). (DOC) [file pone.0130299.s007.doc]

**S2 Table.** Spearman correlation coefficients between environmental variables. Significant correlations are emboldened (significance level is in brackets).

| Variables | Trees | Amenity grass | Arable | Fallow | Meadow | Buildings and roads | Water | Bird feeders | Human Population |
| --- | --- | --- | --- | --- | --- | --- | --- | --- | --- |
| Trees | 1.000 | **0.394** | -0.102 | 0.028 | 0.017 | 0.013 | 0.010 | **0.277** | 0.162 |
|  |  | (-0.004) | (0.471) | (0.842) | (0.904) | (0.929) | (0.942) | (0.047) | (0.250) |
|  |  |  |  |  |  |  |  |  |  |
| Amenity grass |  | 1.000 | **-0.394** | -0.083 | **-0.345** | 0.101 | -0.098 | **0.577** | 0.115 |
|  |  |  | (0.004) | (0.557) | (0.012) | (0.474) | (0.489) | (<0.001) | 0.418 |
|  |  |  |  |  |  |  |  |  |  |
| Arable |  |  | 1.000 | **0.651** | **0.614** | **-0.606** | **0.354** | **-0.397** | -0.120 |
|  |  |  |  | (<0.001) | (<0.001) | (<0.001) | (0.010) | (0.004) | 0.397 |
|  |  |  |  |  |  |  |  |  |  |
| Fallow |  |  |  | 1.000 | **0.344** | **-0.491** | **0.421** | -0.089 | -0.018 |
|  |  |  |  |  | (0.013) | (<0.001) | (0.002) | (0.532) | (0.899) |
|  |  |  |  |  |  |  |  |  |  |
| Meadow |  |  |  |  | 1.000 | **-0.338** | **0.369** | **-0.360** | -0.087 |
|  |  |  |  |  |  | (0.014) | (0.007) | (0.009) | 0.540 |
|  |  |  |  |  |  |  |  |  |  |
| Buildings |  |  |  |  |  | 1.000 | **-0.407** | 0.258 | -0.001 |
| and roads |  |  |  |  |  |  | (0.003) | (0.065) | (0.992) |
|  |  |  |  |  |  |  |  |  |  |
| Water |  |  |  |  |  |  | 1.000 | 0.054 | -0.066 |
|  |  |  |  |  |  |  |  | (0.703) | (0.642) |
|  |  |  |  |  |  |  |  |  |  |
| Bird feeders |  |  |  |  |  |  |  | 1.000 | -0.018 |
|  |  |  |  |  |  |  |  |  | (0.897) |
|  |  |  |  |  |  |  |  |  |  |
| Humam population |  |  |  |  |  |  |  |  | 1.000 |
|  |  |  |  |  |  |  |  |  |  |
|  |  |  |  |  |  |  |  |  |  |
