# Supplementary material for: Winter Bird Assemblages in Rural and Urban Environments: A National Survey
Source: PLoS One. 2015 Jun 18;10(6):e0130299. doi: 10.1371/journal.pone.0130299 (PMC4472663; doi:10.1371/journal.pone.0130299)
Supplement: S3 Table — (DOC) [file pone.0130299.s008.doc]

**S3 Table.** Estimations for random effects in GLMMs

| Dependent variable | Random effect | Estimate | SE | *Z* | *P* |
| --- | --- | --- | --- | --- | --- |
| Corrected number of species (Chao estimator) | Observer | 9.045 | 5.234 | 1.728 | 0.084 |
|  | **Plot identity** | 6.897 | 2.194 | 3.143 | 0.002 |
|  | Town identity | 0.219 | 2.268 | 0.097 | 0.923 |
| Corrected number of birds (Royle estimator) | Observer | 0.216 | 0.248 | 0.872 | 0.383 |
|  | **Plot identity** | 0.287 | 0.061 | 4.739 | <0.001 |
|  | Town identity | 0.163 | 0.205 | 0.796 | 0.426 |
| Diversity index (calculated on corrected numbers) | Observer | 0.000* |  |  |  |
|  | **Plot identity** | 0.015 | 0.002 | 7.250 | <001 |
|  | Town identity | 0.001 | 0.001 | 0.364 | 0.716 |

* variable not contributing to variance
