# Supplementary material for: Winter Bird Assemblages in Rural and Urban Environments: A National Survey
Source: PLoS One. 2015 Jun 18;10(6):e0130299. doi: 10.1371/journal.pone.0130299 (PMC4472663; doi:10.1371/journal.pone.0130299)
Supplement: S4 Table — The Akaike information criterion score (AICc), the -2log, difference between the given model and the most parsimonious model (Δ) and the Akaike weight (w) are listed. Explanation of variable codes: Month—month of survey (December vs. January), Environment—type of environment (urban vs. rural), Longitude—geographical longitude, Latitude—geographical latitude, PCA1—a first principal component of environmental variables describing the increasing cover of open agricultural habitats, PCA2—a second principal component of environmental variables describing the gradient from natural grasslands (meadows) to intensively managed amenity grassland, Feeders—number of bird feeders in a square plot, CitySize—human population size in the city. The best model is emboldened. (DOC) [file pone.0130299.s009.doc]

**S4 Table.** Generalized linear mixed models (GLMM) describing the species richness of birds in urban and rural areas during the winter. The Akaike information criterion score (AICc), the -2log, difference between the given model and the most parsimonious model (Δ) and the Akaike weight (*w*) are listed. Explanation of variable codes: Month – month of survey (December vs. January), Environment – type of the environment (urban vs. rural), Longitude – geographical longitude, Latitude – geographical latitude, PCA 1 – a first principal component of environmental variables describing the increasing cover of open agricultural habitats, PCA2 – a second principal component of environmental variables describing the gradient from natural grasslands (meadows) to the intensively managed amenity grassland, Feeders – number of bird feeders in a square plot, CitySize – human population size in the city. The best model is emboldened.

| Model | AICc | -2log | Δ | *w* |
| --- | --- | --- | --- | --- |
| **Month** | **219.386** | **211.255** | **0.000** | **0.934** |
| Month+Longitude | 224.998 | 216.867 | 5.612 | 0.056 |
| Month+Longitude+Latitude | 228.884 | 220.752 | 9.498 | 0.008 |
| Environment+Month+Longitude+Latitude | 232.817 | 224.684 | 13.431 | 0.001 |
| Intercept only^a^ | 234.773 | 226.643 | 15.387 | 0.000 |
| Environment | 238.708 | 230.576 | 19.322 | 0.000 |
| PCA1 | 238.782 | 230.651 | 19.396 | 0.000 |
| Environment+Month+Longitude+Latitude+PCA1 | 238.845 | 230.712 | 19.459 | 0.000 |
| CitySize | 239.191 | 231.060 | 19.805 | 0.000 |
| PCA2 | 239.957 | 231.826 | 20.571 | 0.000 |
| Feeders | 240.456 | 232.325 | 21.070 | 0.000 |
| Latitude | 241.406 | 233.274 | 22.020 | 0.000 |
| Longitude | 242.579 | 234.448 | 23.193 | 0.000 |
| Environment+Month+CitySize+Longitude+Latitude+PCA1 | 243.436 | 235.303 | 24.050 | 0.000 |
| Environment+Month+CitySize+Longitude+Latitude+PCA1+PCA2 | 249.180 | 241.046 | 29.794 | 0.000 |
| Environment+Month+CitySize+Feeders+Longitude+Latitude+PCA1+PCA2 | 254.803 | 246.669 | 35.417 | 0.000 |
| Environment+Month+CitySize+Feeders+Longitude+Latitude+PCA1+PCA2+Environment*PCA2 | 257.731 | 249.596 | 38.345 | 0.000 |
| Environment+Month+CitySize+Feeders+Longitude+Latitude+PCA1+PCA2+Environment*Month+Environment *PCA2 | 259.299 | 251.164 | 39.913 | 0.000 |
| Environment+Month+CitySize+Feeders+Longitude+Latitude+PCA1+PCA2+Environment*Month+Environment *Latitude+Environment*PCA2 | 263.936 | 255.801 | 44.550 | 0.000 |
| Environment+Month+CitySize+Feeders+Longitude+Latitude+PCA1+PCA2+Environment*Month+Environment *CitySize+Environment*Latitude+Environment*PCA2 | 267.757 | 259.621 | 48.371 | 0.000 |
| Environment+Month+CitySize+Feeders+Longitude+Latitude+PCA1+PCA2+Environment*Month+Environment *CitySize+Environment*Feeders+Environment*Latitude+Environment*PCA2 | 272.322 | 264.185 | 52.936 | 0.000 |
| Environment+Month+CitySize+Feeders+Longitude+Latitude+PCA1+PCA2+Environment*Month+Environment *CitySize+Environment*Feeders+Environment*Longitude+Environment*Latitude+Environment*PCA2 | 278.507 | 270.370 | 59.121 | 0.000 |
| Environment+Month+CitySize+Feeders+Longitude+Latitude+PCA1+PCA2+Environment*Month+Environment *CitySize+Environment*Feeders+Environment*Longitude+Environment*Latitude+Environment*PCA1+ Environment*PCA2 | 283.567 | 275.430 | 64.181 | 0.000 |

^a^ - a null model with the intercept only
