# Supplementary material for: Winter Bird Assemblages in Rural and Urban Environments: A National Survey
Source: PLoS One. 2015 Jun 18;10(6):e0130299. doi: 10.1371/journal.pone.0130299 (PMC4472663; doi:10.1371/journal.pone.0130299)
Supplement: S7 Table — (DOC) [file pone.0130299.s012.doc]

**S7 Table.** Probability of detection for species in urban and rural landscapes derived from Kendall and Royle estimators (Royle 2004; Kendal et al. 2013).

|  | Royle estimator | | | Kendall estimator | | |
| --- | --- | --- | --- | --- | --- | --- |
| Species | rural | urban | total | rural | urban | total |
| *Accipiter gentilis* | - | - | 0.138 (0.013- 0.668) | - | - | 0.336 |
| *Accipiter nisus* | 0.1659 (0.0280 - 0.5787) | 0.0003 (0.0002 - 0.0006) | 0.132 (0.026-0.467) | 0.629 | 0.116 | 0.454 |
| *Aegithalos caudatus* | - | - | 0.0011 (0.0002 - 0.0077) | - | **-** | 0.058 |
| *Anas* platyrhynchos | 0.687 (0.542 - 0.803) | 0.096 (0.037 - 0.226) | 0.562 (0.431 - 0.685) | 0.779 | 0.733 | 0.729 |
| *Ardea cinerea* | - | - | 0.500 (0.123 - 0.876) | - | - | 0.891 |
| *Asio otus* |  |  |  |  |  |  |
| *Bombycilla garrulus* | 0.108 (0.071 - 0.162) | 0.148 ( 0.134 - 0.163) | 0.119 (0.106 - 0.133) | 0.523 | 0.543 | 0.546 |
| *Buteo buteo* | - | - | - | **-** | - | - |
| *Buteo lagopus* | - | - | - | - | - | - |
| *Carduelis cannabina* | 0.514 (0.514 - 0.514) | - | 0.4742 ( 0.4739 - 0.4745) | - | - | 0.806 |
| *Carduelis carduelis* | 0.009 (0.005 - 0.016) | 0.0010 (0.0001 - 0.0182) | 0.06 0.03 - 0.11) | 0.473 | - | 0.359 |
| *Carduelis flammea* | - | - | - | - | - | - |
| *Carduelis flavirostris* | - | - | - | - | - | - |
| *Carduelis spinus* | 0.115 (0.078 - 0.166) | 0.119 (0.048- 0.264) | 0.123 (0.089 - 0.168) | 0.658 | - | 0.696 |
| *Certhia brahydactyla* | - | - | *-* | - | - | - |
| *Certia familaris* | - | - | - | - | - | - |
| *Chloris chloris* | 0.180 ( 0.141 - 0.227) | 0.201 ( 0.163 - 0.246) | 0.192 (0.164 - 0.224) | 0.832 | 0.851 | 1.000 |
| *Chroicocephalus ridibundus* | *1* | 0.604 (0.566 - 0.641) | 0.719 (0.688 -0.749) | 1 | 0.937 | 0.903 |
| *Coccothraustes coccothraustes* | *0.522* | 0.051 (0.003 - 0.501) | 0.100 (0.038 - 0.241) | 0.834 | - | - |
| *Columba livia* | 0.379 (0.379 - 0.380) | 0.609 (0.594 - 0.625) | 0.652 (0.637 - 0.667) | 0.926 | 1 | 0.985 |
| *Columba palumbus* | 0.377 (0.161- 0.653) | 0.621 (0.506 - 0.724) | 0.591 (0.486 - 0.688) | 0.645 | 0.798 | 0.767 |
| *Corvus corax* | 0.443 (0.235 - 0.674) | - | 0.449 (0.251 - 0.665) | 0.755**-** | - | 0.752 |
| *Corvus cornix* | 0.368 (0.225 - 0.539) | 0.492 (0.397 - 0.588) | 0.475 (0.396 - 0.556) | 0.702 | 0.908 | 0.875 |
| *Corvus corone* | - | - | - | - | - | - |
| *Corvus frugilegus* | 0.545 (0.511 - 0.579) | 0.487 (0.469 - 0.504) | 0.513 (0.495 - 0.531) | 0.884 | 1.000 | 1.000 |
| *Corvus monedula* | 0.514 (0.458 - 0.569) | 0.494 (0.469 - 0.519) | 0.588 (0.567 - 0.609) | 0.844 | 1.000 | 1 |
| *Cyanistes caeruleus* | 0.460 0.340 - 0.543) | 0.272 (0.199 - 0.362) | 0.355 (0.298 - 0.416) | 1 | 1.000 | 1.000 |
| *Dendrocopos major* | 0.323 (0.176 - 0.516) | 0.157 (0.026 - 0.566) | 0.309 (0.187 - 0.465) | 0.662 | 0.529 | 0.738 |
| *Dendrocopos medius* | - | - | - | - | - | - |
| *Dendrocopos minor* | - | - | - | - | - | - |
| *Dendrocopos syriacus* | - | - | 0.218 (0.046 - 0.617) | - | - | 0.503 |
| *Dryocopus martius* | - | - | - | - | - | - |
| *Emberiza calandra* | - | - | 0.085 (0.002 - 0.644) | - | - | 0.582 |
| *Emberiza citrinella* | 0.124 (0.079 - 0.190) | 0.0015 (0.0002 - 0.0114) | 0.171 (0.124 - 0.229) | 0.755 | 0.053 | 0.730 |
| *Emberiza schoeniclus* | - | - | - | - | - | - |
| *Erithacus rubecula* | 0.160 (0.048 - 0.415) | 0.084 (0.004 - 0.695) | 0.157 (0.061 - 0.350) | 0.717 | 0.527 | 0.672 |
| *Falco tinnunculus* | - | 0.219 (0.023 - 0.769) | 0.123 (0.010 - 0.661) | - | 0.289 | 0.455 |
| *Fringilla coelebs* | 0.046 (0.003 - 0.458) | 0.086 (0.020 - 0.301) | 0.067 (0.019 - 0.216) | 0.761 | 0.725 | 0.728 |
| *Fringilla montifringilla* | 0.0971 (0.007 - 0.606) | - | 0.103 (0.011 - 0.543) | 0.594 | - | 0.582 |
| *Galerida cristata* | - | - | 0.856 (0.545 - 0.967) | - | - | - |
| *Garrulus glandarius* | 0.405 (0.299- 0.520) | *0.4145 (0.246 - 0.607)* | 0.456 (0.370 - 0.546) | 0.853 | 0.717 | 0.865 |
| *Haliaeetus albicilla* | - | - | - |  | - | - |
| *Lanius excubitor* | - | - | - | - | - | - |
| *Larus argentatus* | - | 0.623 (0.547 - 0.693) | 0.640 (0.569 - 0.704) | - | 0.981 | 0.829 |
| *Larus canus* | - | 0.721 (0.679 - 0.759) | 0.671 (0.596- 0.738) | - | 0.996 | 0.868 |
| *Lophophanes cristatus* | 0.151 (0.030 - 0.504) | 0.562 (0.170 - 0.889) | 0.243 (0.092 - 0.504) | 0.498 | 0.612 | 0.482 |
| *Loxia curvirostra* | - | - | - | - | - | - |
| *Poecile montanus* | 0.286 (0.070- 0.681) | - | 0.297 (0.079 - 0.677) | 0.544 |  | 0.511 |
| *Poecile palustris* | 0.332 (0.179 - 0.530) | - | 0.311 (0.175 - 0.489) | 0.670 | - | 0.525 |
| *Parus major* | 0.427 (0.427 - 0.428) | 0.475 (0.431 - 0.521) | 0.483 (0.452 - 0.514) | 1.000 | 1.000 | 1.000 |
| *Passer domesticus* | 0.568 (0.549 - 0.588) | 0.709 (0.689 - 0.728) | 0.631 (0.617 - 0.645) | 1.000 | 1.000 | 1.000 |
| *Passer montanus* | 0.428 (0.390 - 0.466) | 0.429 (0.381 - 0.478) | 0.438 (0.408 - 0.467) | 0.875 | 0.908 | 0.995 |
| *Periparus ater* | 0.273 (0.154 - 0.436) | 0.122 (0.010 - 0.661) | 0.283 (0.174 - 0.425) | 0.604 | 1 | 0.5992 |
| *Perdix perdix* | - | - | - | - | - | - |
| *Phalacrocorax carbo* | - | - | - | - | - | - |
| *Phasianus colchicus* | 0.187 (0.043 - 0.539) |  | 0.215 (0.064 - 0.522) | 0.586 |  | 0.562 |
| *Phoenicuros ochruros* | - | - | - | - | - | - |
| *Pica pica* | 0.459 (0.384 - 0.535) | 0.572 (0.518 - 0.625) | 535 (0.489 - 0.577) | 1.000 | 0.932 | 1.000 |
| *Picus viridis* | - | - | - | - | - | - |
| *Prunella modularis* | - | - | - | - | - | - |
| *Pyrhulla pyrhulla* | 0.287 (0.181 - 0.423) | 0.185 (0.081 - 0.365) | 0.259 (0.178 - 0.362) | 0.670 | 0.726 | 0.719 |
| *Regulus regulus* | 0.215 (0.093 - 0.423) | 0.185 (0.039 - 0.555) | 0.233 (0.123 - 0.398) | 0.687 | 0.381 | 0.523 |
| *Serinus serinus* | - | - | - | - | - | - |
| *Sitta europea* | 0.354 (0.181 - 0.576) | 0.098 (0.008 - 0.601) | 0.275 (0.143 - 0.463) | 0.621 | 0.343 | 0.431 |
| *Streptopelia decaocto* | 0.529 (0.528 - 0.529) | 0.523 (0.467- 0.578) | 0.428 (0.427 - 0.428) | 0.961 | 0.963 | 1.000 |
| *Sturnus vulgaris* | - | 0.374 (0.174 - 0.629) | 0.351 (0.164 - 0.598) | - | 0.409 | 0.365 |
| *Troglodytes troglodytes* | - | 0.497 (0.080 - 0.918) | 0.183 (0.037 - 0.561) | - | 1.000 | 0.708 |
| *Turdus iliacus* | - | - | - | - | - | - |
| *Turdus merula* | 0.411 (0.347 - 0.477) | 0.391 (0.333 - 0.453) | 0.401 (0.357 - 0.445) | 0.986 | 0.940 | 1.000 |
| *Turdus philomelos* | - | - | - | - | - | - |
| *Turdus pilaris* | 0.351 (0.317- 0.440) | 0.448 (0.391 - 0.532) | 0.388 (0.355 - 0.441) | 0.792 | 0.766 | 0.990 |
| *Turdus viscivorus* | - | - | - | - | - | - |
